# Vitamin D Intake and Risk of Type 1 Diabetes: A Meta-Analysis of Observational Studies

**DOI:** 10.3390/nu5093551

**Published:** 2013-09-12

**Authors:** Jia-Yi Dong, Weiguo Zhang, Jiong Jack Chen, Zeng-Li Zhang, Shu-Fen Han, Li-Qiang Qin

**Affiliations:** 1 Department of Nutrition and Food Hygiene, School of Public Health, Soochow University, 199 Renai Road, Suzhou 215123, China; E-Mails: dongjy@mail3.sysu.edu.cn (J-Y.D.); sfhan@suda.edu.cn (S-F.H.); 2 DSM Nutritional Products, Human Nutrition and Health, No.1-3 Xinyuannanlu, Beijing 100027, China; E-Mail: weiguo.zhang@dsm.com; 3 DSM Nutritional Products, Research Center, 476 Libing Road, Shanghai 201203, China; E-Mail: jack.chen@dsm.com; 4 Department of Labor Hygiene and Environmental Health, School of Public Health of Soochow University, 199 Renai Road, Suzhou 215123, China; E-Mail: zhangzengli@suda.edu.cn

**Keywords:** vitamin D, type 1 diabetes, early life, pregnancy, meta-analysis

## Abstract

Vitamin D is suggested to have protective effects against type 1 diabetes. However, the results from observational studies have been inconsistent. We aimed to examine their association by conducting a meta-analysis of observational studies. Multiple databases were searched in June 2013 to identify relevant studies including both case-control and cohort studies. Either a fixed- or random-effects model was used to calculate the pooled risk estimate. We identified eight studies (two cohort studies and six case-control studies) on vitamin D intake during early life and three studies (two cohort studies and one case-control study) on maternal vitamin D intake during pregnancy. The pooled odds ratio for type 1 diabetes comparing vitamin D supplementation with non-supplementation during early life was 0.71 (95% confidence interval [CI], 0.51–0.98). Similar results were observed in the case-control subgroup analysis but not in the cohort subgroup analysis. The pooled odds ratio with maternal intake of vitamin D during pregnancy was 0.95 (95% CI, 0.66–1.36). In conclusion, vitamin D intake during early life may be associated with a reduced risk of type 1 diabetes. However, there was not enough evidence for an association between maternal intake of vitamin D and risk of type 1 diabetes in the offspring.

## 1. Introduction

Type 1 diabetes is an immune-mediated disease characterized by the loss of the insulin-producing β-cells, which eventually results in high levels of glucose in the blood. The incidence of type 1 disease is increasing all over the world [1,2]. Ecological study has shown an association between ultraviolet B irradiance, the primary source of circulating vitamin D in humans, and high incidence of type 1 diabetes in children, leading to the hypothesis that vitamin D may have a role in the disease [3]. The prevalent vitamin D deficiency/insufficiency, for example in the Chinese population, represents an important public health issue [4].

Indeed, recent cross-sectional studies consistently suggested a high vitamin D deficiency in patients with type 1 diabetes [5,6,7]. EURODIAB, the first case-control study on this topic, showed a reduced risk of developing type 1 diabetes by vitamin D supplementation during the first year of life [8]. However, subsequent observational studies (case-control and cohort studies) yielded inconsistent results [9,10,11,12,13,14,15]. The protective effect of vitamin D against type 1 diabetes was observed in some studies [8,10,11,13,14] but not all [9,12,15]. The small sample size may have led to the null associations in some studies. On the other hand, there is evidence that the destruction of β-cells could be initiated in utero [16]. Growing attention therefore has been paid to the role of maternal vitamin D status in type 1 diabetes in the offspring. Yet the results from observational studies [9,11,17] were also inconclusive.

Thus, the aim of this study was to examine the association between vitamin D intake and risk of type 1 diabetes by conducting a meta-analysis of relevant studies. In addition, we examined the association between maternal intake of vitamin D during pregnancy and risk of type 1 diabetes in the offspring.

## 2. Methods

### 2.1. Literature Search

We generally followed the Meta-Analysis of Observational Studies in Epidemiology (MOOSE) guidelines [18] in the design, analysis, and reporting of this study. We searched PubMed, Web of Science, and Cochrane Library databases in June 2013 to identify relevant studies that assessed the association between intake of vitamin D and risk of type 1 diabetes. The literature search was supplemented through the manual review of reference lists of retrieved articles and recent reviews. Search terms included “vitamin D”, “25-hydroxyvitamin D”, “type 1 diabetes”. No restriction was imposed.

### 2.2. Study Selection

Studies were eligible for analysis if they met the following criteria: the study design was a case-control study or cohort study; the outcome was type 1 diabetes; and risk estimates of diabetes related to vitamin D intake during early life or maternal intake of vitamin D and associated 95% CIs (or data to calculate them) were reported. Initially, we reviewed titles and abstracts to ascertain the potential fit to the inclusion criteria. In the presence of uncertainty regarding the relevancy, a subsequent full-text assessment was conducted.

### 2.3. Data Extraction

We extracted the following information: the first author’s name, publication year, study population, location, characteristics of the study population (age, sex, and sample size), assessment of exposure and outcome, length of follow-up (for cohort studies), most fully adjusted risk estimates (with corresponding 95% CI) from a multivariable model, and statistical adjustment for the potential confounding factors. For two studies [15,17] that examined different sources of vitamin D (*i.e.*, total vitamin D, vitamin D from food only, and vitamin D from supplementation only), the risk estimates for total vitamin D intake were used. Two authors (Dong, J.Y. and Qin, L.Q.) independently performed data extraction, with disagreements resolved by discussion.

### 2.4. Statistical Analysis

Odds ratio (OR) was used as a measure of the association between vitamin D intake and risk of type 1 diabetes. For cohort studies, incidence rate, rate ratio, and relative risk were considered as a surrogate measure of OR. Because the absolute risk of type 1 diabetes is low, the relative risks approximate OR values. We calculated the *Q* and *I^2^* statistics [19] to examine statistical heterogeneity across studies. *I^2^* is the proportion of total variation contributed by between-study variation. Either a fixed-effects model or, in the presence of heterogeneity, random-effects model was used to combine the study-specific ORs and compute the summary one. Subgroup analysis by study design (*i.e.*, case-control *vs.* cohort studies) was performed. We also conducted sensitivity analysis to investigate the influence of a single study on the overall risk estimates by omitting one study in each turn. Potential publication bias was assessed by Egger test [20]. All analyses were performed using STATA version 11.0 [21]. A *P* value <0.05 was considered statistically significant.

## 3. Results

### 3.1. Literature Search

**Figure 1:**
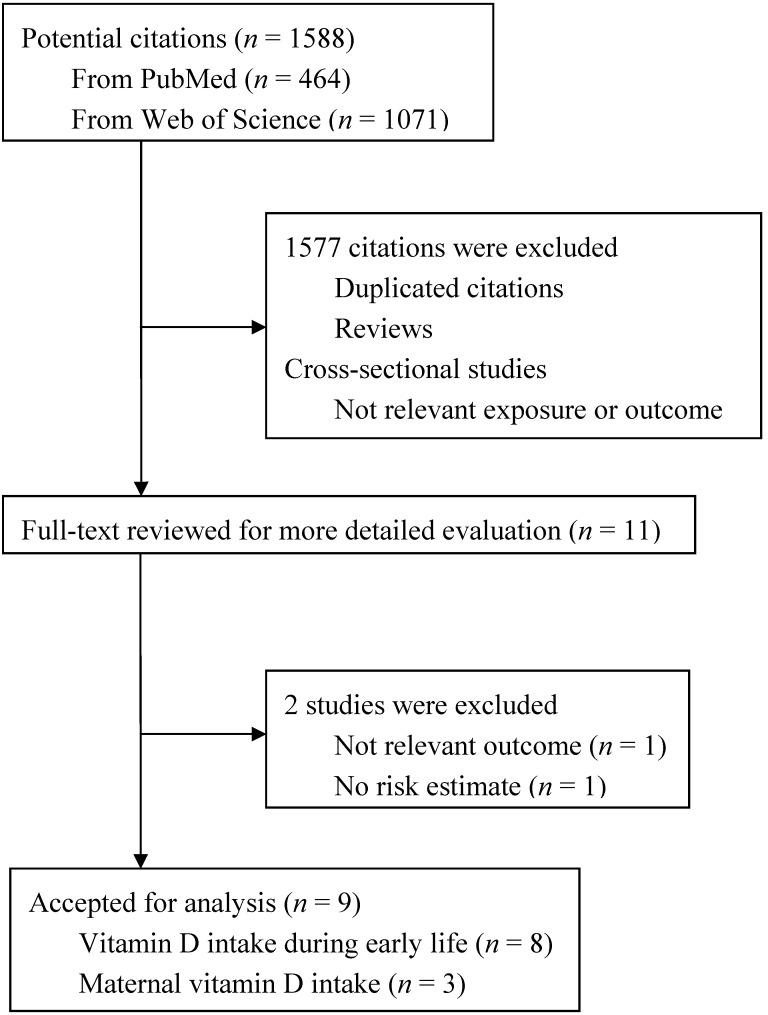
Flow chart of literature search and study selection.

Figure 1 presents a flow chart showing the study selection process. We initially identified 1588 potential articles from three electronic databases; most were excluded because they were not case-control or cohort studies or because the exposure or endpoint was not relevant to our analysis, leaving 11 potentially eligible papers for full-text review. One study [22] was excluded because the outcome was immune-mediated diseases but not type 1 diabetes. Another study [23] was excluded because the risk estimate for the association of interest was not available. Finally, nine articles [8,9,10,11,12,13,14,15,17] with 12 risk estimates on vitamin D and type 1 diabetes were included in the final analysis. The EURODIAB [8] that combined the results of seven case-control studies across European countries was considered as one multi-center, case-control study. Among the nine articles, eight studies [8,9,10,11,12,13,14,15] reported results on vitamin D intake during early life (e.g., the first year of life or childhood period) and three studies [9,11,17] reported results on maternal vitamin D intake during pregnancy.

### 3.2. Study Characteristics

Characteristics of the selected studies are presented in Table 1. These studies were published between 1999 and 2011. Among them, seven studies were conducted in Europe, and one each in the United States and Iran. Six studies had a case-control design, comprising 1860 cases and 6243 controls; the other three had a cohort design, comprising 171 cases and 14,264 participants. The length of follow-up for cohort studies ranged from 5 to 31 years. Most selected/incident cases aged ≤ 18 years old, whereas two studies had cases with a wider age range between 0 and 30 years old. All studies included both male and female participants. Two studies used food-frequency questionnaires for assessment of vitamin D intake and the remaining used other questionnaires or interview. Type 1 diabetes cases were mainly ascertained on the basis of WHO criteria or EURODIAB criteria. The adjusted covariates in individual studies differed from each other.

**Table 1:** Characteristics of observational studies on vitamin D intake and risk of type 1 diabetes.

Study	Design	Age at Diagnosis (years)	Sex	No. of Cases/No. of Control (Participants)	Exposure Comparison	Exposure Assessment	Case Ascertainment	Adjustment
EURODIAB study [8], Europe	Case-control	<15	M/F	820/2335	Vitamin D supplementation during early infancy (yes *vs.* no)	Questionnaire, interview	EURODIAB criteria	Duration of breast feeding less than 3 months, maternal age over 35 years, birth weight less than 2500 g, and study center
Stene *et al.* [9], Norway	Case-control	<15	M/F	85/1071	Cod liver oil intake during pregnancy/during first year of life (yes *vs.* no)	Questionnaire	EURODIAB criteria	Age, sex, breastfeeding, maternal education, and other supplement
Hypponen *et al.* [10], Finland	Birth-cohort (1966–1997)	1–31	M/F	81/10366	Vitamin D supplementation during the first year (regular *vs.* none)	Questionnaire	Hospital discharge registers, or medical records	Sex, neonatal, length of maternal education, social status, birth weight, and growth rate in infancy
Stene *et al.* [11], Norway	Case-control	<15	M/F	545/1668	Cod liver oil intake during pregnancy/during first year of life (yes *vs.* no)	Questionnaire	EURODIAB criteria	Age, sex, duration of exclusive breastfeeding, age at introduction of solid foods, maternal education, maternal smoking, maternal age at delivery, childʼs number of siblings, type 1 diabetes among childʼs siblings or parents
Visalli *et al.* [12], Italy	Case-control	6–18	M/F	150/750	Vitamin D supplementation during early life (yes *vs.* no)	Questionnaire	EURODIAB criteria	Not available
Tenconi *et al.* [13], Italy	Case-control	0–29	M/F	159/318	Vitamin D supplementation during lactation (yes *vs.* no)	Interview	Not available	Age, sex, viral diseases, bottle feeding, scarlet fever
Ahadi *et al.* [14], Iran	Case-control	<16	M/F	101/101	Vitamin D supplementation during the first year(yes *vs.* no)	Questionnaire	WHO criteria	Maternal age at delivery, type of delivery, type of feeding
Simpson *et al.* [15], USA	Cohort (1993–2011)	<12	M/F	35/175	Vitamin D supplementation during childhood (yes *vs.* no)	Food-frequency questionnaires	Physician diagnosis	Family history, genotype, energy intake, survey type, and age at first appearance of auto-antibodies
Marjamaki *et al.* [17], Finland	Birth-cohort (1997–2002)	1-8	M/F	55/3723	Total vitamin D intake during pregnancy (yes *vs.* no)	Food-frequency questionnaires	WHO criteria	Genetic risk, familial type 1 diabetes, sex, gestational age, maternal age, maternal education, delivery hospital, route of delivery, number of earlier deliveries and smoking during pregnancy

### 3.3. Vitamin D Intake and Risk of Type 1 Diabetes

**Figure 2:**
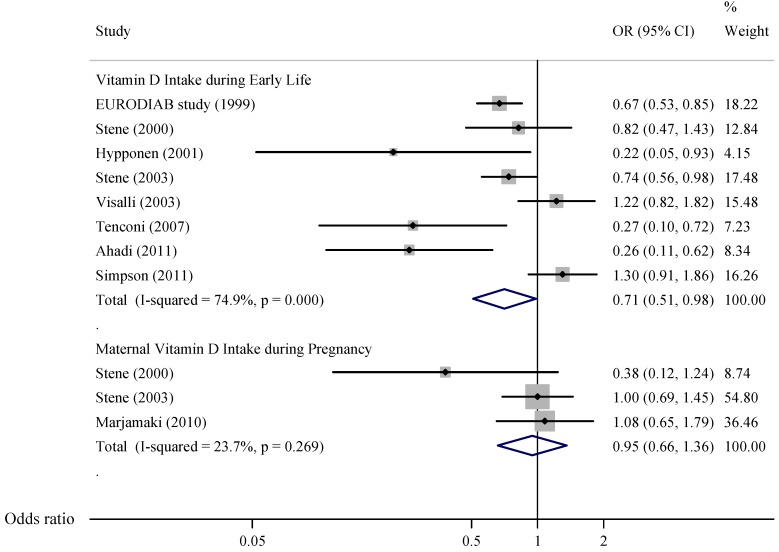
Meta-analysis of observational studies on vitamin D intake and risk of type 1 diabetes.

There were eight epidemiologic studies (six case-control studies and two cohort studies) reporting the association between vitamin D intake and risk of type 1 diabetes. All of them focused on vitamin D intake during early life. The ORs from individual studies varied from 0.22 to 1.30. Of the eight included studies, five reported a significant inverse association between vitamin D intake and risk of type 1 diabetes, while the other three found null associations. The pooled OR was 0.71 (95% CI, 0.51–0.98; Figure 2), with evidence of heterogeneity across studies (*P* < 0.001, *I^2^* = 74.9%). We further conducted subgroup analysis by study design (case-control studies *vs.* cohort studies). The pooled risk estimate for six case-control studies was similar to the overall one (OR = 0.68; 95% CI, 0.49–0.94), while the pooled risk estimate for two cohort studies was non-significant with a wide CI (relative risk = 0.62; 95% CI, 0.11–3.45). The sensitivity analysis in which one study at a time was omitted generally showed statistically significant or marginally significant results; the pooled ORs ranged from 0.64 (95% CI, 0.46–0.89) to 0.78 (95% CI, 0.57–1.07). The Egger test suggested no evidence of publication bias (*P* = 0.28).

Three studies (two case-control studies and one cohort study) examined the association between maternal intake of vitamin D and risk of type 1 diabetes in the offspring. All of them reported non-significant associations. The pooled OR was 0.95 (95% CI, 0.66–1.36; Figure 2), with little evidence of heterogeneity (*P* = 0.27, *I^2^* = 23.7%). Because of the small number of studies, subgroup analysis, sensitivity analysis, and Egger test were not performed.

## 4. Discussion

The present meta-analysis aimed to assess the association between vitamin D intake and risk of type 1 diabetes. By combining the results from published case-control and cohort studies to date, our findings suggested vitamin D intake during early life was associated with a reduced risk of type 1 diabetes. However, current evidence does not support an association between maternal intake of vitamin D and risk of type 1 diabetes in the offspring.

Complex mechanisms (which are beyond our scope) may be involved in the potentially protective effects of vitamin D against type 1 diabetes. The beneficial effects of vitamin D are proposed to be related to the regulation of the immune system, including its actions in monocytes, macrophages, dendritic cells, T and B lymphocytes. In fact, experimental studies and clinical trials have shown beneficial effects of vitamin D supplementation on immune function [24]. Another possible mechanism is that vitamin D may have direct effects on β-cells, including improving insulin secretion, enhancing expression of vitamin D receptor, and improving islet morphology [24,25]. In addition, vitamin D may have effects on regulation of inflammatory responses [24].

To date, no randomized controlled trials directly look at the effects of vitamin D supplementation on incidence of type 1 diabetes. Several trials tested the effects of vitamin D supplementation on residual β-cell function in recent-onset type 1 diabetes [26,27,28,29]. However, the results were somewhat mixed. Three trials [26,27,28] reported that calcitriol (the active form of vitamin D) had no protective effects or only modest effects on β-cell function, while one trial [29] suggested cholecalciferol (vitamin D3) used as adjunctive therapy with insulin slowed decline of residual β-cell function. Interestingly, in one trial conducted in healthy subjects, vitamin D3 supplementation did not improve β-cell function but significantly increased the frequency of regulatory T cells [30]. Such protective immunologic effect, which was also observed in an above mentioned trial conducted in type 1 diabetes [29], indicated a possible mechanistic link between vitamin D and the disease.

Measurement of vitamin D intake using questionnaires is inaccurate due to recall bias and other measurement errors; moreover, sun exposure was not taken into account. Therefore, some studies assessed blood 25-hydroxyvitamin D [25(OH)D] level as a reliable measure of total vitamin D status. One case-cohort study found plasma 25(OH)D level was not associated with type 1 diabetes risk in islet autoimmunity-positive children (adjusted hazard ratio = 0.91, 95% CI 0.68–1.22) [15]. The small number of identified cases (*n* = 35) may be responsible for the null association. More recently, one nested case-control study among US military personnel with more cases (*n* = 310) reported that higher blood 25(OH)D levels was associated with a significantly lower risk of type 1 diabetes among non-Hispanic whites, although such association was not observed in non-Hispanic blacks or Hispanics [31].

Whether vitamin D deficiency in the fetal period contributes to increased risk of type 1 diabetes is of additional interest. Previous study indicated maternal intake of vitamin D had a protective effect on preventing diabetes-associated autoimmunity in the children [32]. Yet, our meta-analysis of three studies [9,11,17] provided no evidence for the association between maternal intake of vitamin D and diabetes risk in the offspring. In line with our finding, one Finnish study also found no difference in serum 25(OH)D levels during early pregnancy between case and control mothers [33]. By contrast, another study in Norway suggested lower maternal level of 25(OH)D during pregnancy was associated with a significantly higher risk of type 1 diabetes in the offspring (OR = 2.38, 95% CI 1.12–5.07) [34]. Given the limited and inconsistent evidence at present, more studies are needed to determine the role of vitamin D in the fetal period in the development of type 1 diabetes.

Compared with a previous meta-analysis of only five studies [35], we added more evidence linking vitamin D and type 1 diabetes up to date. We were also able to examine the association between maternal vitamin D intake during pregnancy and diabetes risk in the offspring, which was not measured in that meta-analysis. However, limitations of the present study should be considered. First, because the findings were mainly based on case-control studies, recall bias and selection bias were inevitable. Also, the adjusted covariates in individual studies differed and the lack of control for important confounding factors increased the risk of confounding bias. Second, there was considerable heterogeneity among studies. Study design did not seem to explain much between-study variation as heterogeneity remained in both case-control and cohort subgroups (data not shown). Third, measurement errors could influence our findings as assessments of vitamin D intake largely depended upon questionnaires. Moreover, whether the questionnaires were validated was not reported in several studies. Fourth, we included two studies [9,11] using cod liver oil as vitamin D supplementation, we therefore could not rule out the possibility that *n*-3 fatty acids in cod liver oil may, at least in part, account for the observed benefits on type 1 diabetes risk. Finally, although there was no evidence of publication bias, we could not completely rule out such bias because of the limited number of studies.

## 5. Conclusions

Our findings suggest that vitamin D intake during early life is associated with a reduced risk of type 1 diabetes. There was not enough evidence supporting an association between maternal intake of vitamin D and type 1 diabetes risk in the offspring. Because these findings were largely based upon case-control studies, well-designed cohort studies are still needed to determine the role of vitamin D in the prevention of type 1 diabetes.
